# An organismal perspective on *C. intestinalis* development, origins and diversification

**DOI:** 10.7554/eLife.06024

**Published:** 2015-03-25

**Authors:** Matthew J Kourakis, William C Smith

**Affiliations:** Department of Molecular, Cellular and Developmental Biology, University of California, Santa Barbara, Santa Barbara, United States; Department of Molecular, Cellular and Developmental Biology, University of California, Santa Barbara, Santa Barbara, United States

**Keywords:** the natural history of model organisms, *C. intestinalis*, chordate, tunicate, *C. intestinalis*

## Abstract

The ascidian *Ciona intestinalis*, commonly known as a ‘sea squirt’, has become an important model for embryological studies, offering a simple blueprint for chordate development. As a model organism, it offers the following: a small, compact genome; a free swimming larva with only about 2600 cells; and an embryogenesis that unfolds according to a predictable program of cell division. Moreover, recent phylogenies reveal that *C. intestinalis* occupies a privileged branch in the tree of life: it is our nearest invertebrate relative. Here, we provide an organismal perspective of *C. intestinalis*, highlighting aspects of its life history and habitat—from its brief journey as a larva to its radical metamorphosis into adult form—and relate these features to its utility as a laboratory model.

**DOI:** http://dx.doi.org/10.7554/eLife.06024.001

## Introduction

**Figure 1.**
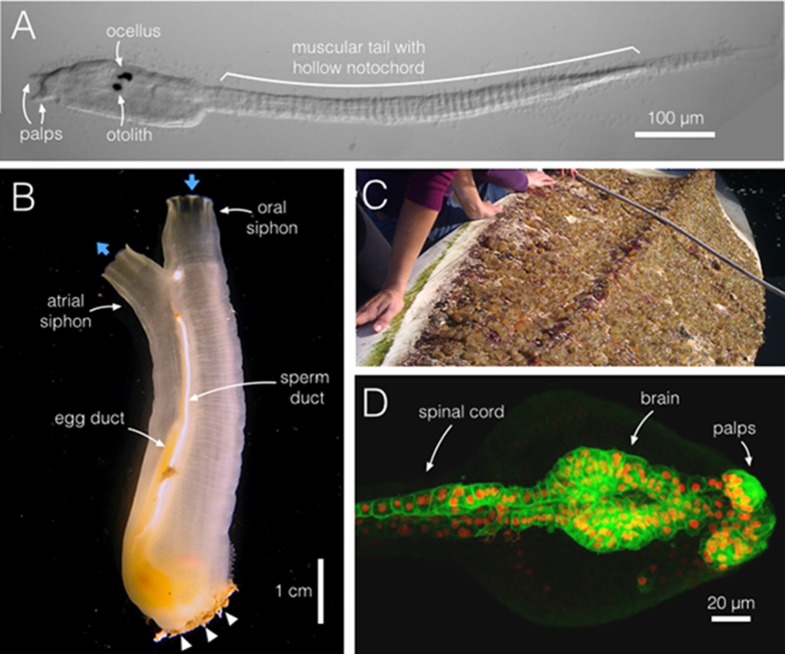
*Ciona intestinalis*, from swimming larva to filter-feeding adult. (**A**) During a brief larval phase, *C. intestinalis* (dorsal is top) finds and attaches to a substrate via its anterior adhesive palps (two of three are shown), where it initiates metamorphosis. The larva swims using a muscular tail, aided by the rigidity and stiffness of the notochord, a hollow tube within the tail. The pigmented brain organs, the ocellus and otolith, which sense light and gravity, help to guide the animal. (**B**) A *C. intestinalis* adult has two siphons, oral and atrial, positioned opposite the attachment point (arrowheads); the flow of water in and out is indicated (blue arrows). *C. intestinalis* are hermaphrodites, and here the egg and sperm ducts are visible; both sperm and eggs exit via the atrial siphon cavity. (**C**) Clusters of *C. intestinalis* attached to the underside of a kayak in Santa Barbara, California. These marine invaders often line vessel hulls and crowd submerged ropes, buoys and other surfaces. (**D**) A confocal projection of the brain and spinal cord of *C. intestinalis* near hatching stage; anterior is right. *C. intestinalis* is ideal for imaging and, unlike its larger chordate cousins, large portions of the animal can be imaged within a single field of view. Cell membranes are in green (*etr>ArcLight*) and nuclei in red (*etr>RFP*). Image credits: (**A**, **B**, **D**), M Kourakis; (**C**), S Abdul-Wajid. **DOI:** http://dx.doi.org/10.7554/eLife.06024.002

The tunicate (sea squirt) *Ciona intestinalis* spends its adult life anchored to a hard substrate, filter feeding and releasing gametes (eggs and sperm) into the surrounding sea water. For a fleeting day or two of its life, however, the larva of *C. intestinalis* adopts a tadpole morphology (Figure 1A). This morphology provides hints as to the origins of the chordates (see Box 1 for a glossary of specialist terms used in this article), including that of the most successful chordate clade, the vertebrates. In 1866, the Russian biologist Alexander Kowalevsky observed the ascidian tadpole and noted the presence of a dorsal nervous system and prominent notochord, two defining features of chordates (Kowalevsky, 1866). Prior to this, the chordate features of tunicates had not been described and, in fact, a century earlier the taxonomy of Carl Linnaeus placed *C. intestinalis* and other ascidians (see Glossary) within the molluscs, based on their adult form (Linné, 1767).

10.7554/eLife.06024.003

Box 1. **Glossary**

**Ascidian**—A common name for a member of the Ascidiacea, a class within the Tunicata. Ascidians include *C. intestinalis* and other models like *Botryllus schlosseri*, *Phallusia mamillata* and *Halocynthia *roretzi**.

**Biofouling**—The accumulation of organisms on underwater surfaces such as pilings, boats, etc.

**Cephalochordate**—These coastal-dwelling fish-like chordates (up to about 7 cm length) are filter feeders and lack vertebra and key head sensory apparatus. They feature somites, highlighting them as a characteristic of the common chordate ancestor, indicating their loss in tunicates.

**Chordate**—This phylum includes tunicates, cephalochordates and vertebrates, and their features include the notochord, dorsal nerve cord, post-anal tail, and a muscular tail or somites. Some investigators consider the endostyle/thyroid to be a shared feature, while pharyngeal gill slits, although shared by all chordates, are not unique to the phylum and are found in related *non*-chordates as well.

**Hox cluster**—An array of Hox genes along a single chromosome. These genes have conserved patterning functions in animal development. Their order within a chromosomal cluster reflects the anterior-posterior order in which they are expressed during development.

**Gastrulation**—A morphological event early in embryogenesis, leading to the formation of ectoderm, mesoderm and endoderm, the precursors of all adult tissues.

**Larvacean**—An order within the tunicates, larvaceans are so-called because of their retention of a larval-like body, most prominently the larval tail, into adult life.

**Neural crest cells**—Multipotent migratory cells in vertebrates that form or contribute to (among other derivatives) specialized sensory and feeding structures, including the jaw and skull, the inner ear and the lateral line sensory system.

**Neurogenic placodes**—Similar to neural crest in their contributions, these structures originate as local thickenings in the embryonic ectoderm. Their derivatives include sensory neurons of the olfactory and auditory systems, and cranial ganglia.

**Neurulation**—The rolling up or hollowing out of tissue in chordates to create a dorsal hollow nerve cord along the anterior-posterior of the developing embryo.

**Operon**—A cluster of genes sharing common transcriptional control, originally identified in bacteria but now known to occur in animals, plants and fungi, as well.

**Somites**—Paired segmental blocks of mesoderm formed during embryogenesis that give rise to skeletal muscle and, in vertebrates, to other structures, including vertebrae and rib cage elements.

**Thaliacean**—A class of free-swimming, ascidian-like tunicates that use their oral and atrial siphons for propulsion through water.

**Tunicate**—The closest living group to vertebrates, the Tunicata subphylum of the chordates is characterized by its ability to make cellulose and by a radical metamorphosis following the larval phase.

**DOI:** http://dx.doi.org/10.7554/eLife.06024.003

Today, due to its unique evolutionary position, and ease of use, *C. intestinalis* is the model organism of choice for many labs that aim to uncover the mechanisms that drive embryonic development and morphogenesis. In the era of recombinant DNA and genomics, many standard tools are available to the *C. intestinalis* researcher, including a well-annotated genome, methods to manipulate gene function and to create mutant strains and reporter lines, and techniques for expressing engineered DNA (Satoh, 2014). Additionally, many resources have been built thanks to the cooperative efforts of *C. intestinalis* investigators (see Box 2).

10.7554/eLife.06024.004

Box 2. **Selected *C. intestinalis* and ascidian resources**

**Aniseed** (http://aniseed.cnrs.fr)—Ascidian Network for In Situ Expression and Embryological Data; provides databases and tools for research on *C. intestinalis* and other ascidians.

**Ascidian Stock Center** (www.ascidiancenter.ucsb.edu)—Provides wild type and stable transgenic *C. intestinalis* and *C. savignyi* to *Ciona* researchers.

**CIPRO** (http://cipro.ibio.jp)—*C. intestinalis* Protein Database.

**Dutch ascidians homepage** (ascidians.com)—Photographs of ascidian species worldwide, showing intra-specific variation.

**FABA** (http://chordate.bpni.bio.keio.ac.jp/faba/1.4/top.html)—Four-dimensional Ascidian Body Atlas; provides high resolution images of *C. intestinalis* during development.

**Ghost Database** (http://ghost.zool.kyoto-u.ac.jp/cgi-bin/gb2/gbrowse/kh/)—Gene expression, genomic and cDNA resources for *C. intestinalis* research.

**Tunicate Meeting** (tunicatemeeting.info)—A biennial forum for tunicate biologists that takes place alternately in Japan, North America or Europe.

**Tunicate Web Portal** (http://www.tunicate-portal.org/wordpress/)—Provides links to tunicate databases and websites, and community information for tunicate researchers.

**DOI:** http://dx.doi.org/10.7554/eLife.06024.004

Unlike researchers working on more common model organisms, those working on *C. intestinalis* rely on wild-caught specimens. The large-scale culturing of *C. intestinalis* is limited to only a few laboratories that have marine facilities, and attempts to establish an inbred laboratory strain of *C. intestinalis* for widespread use have not yet been successful. Researchers should be aware that differences between isolates (for example, in their geography, ecology, and life history), while potentially confounding, might also lead to new insights regarding regional adaptation and variation, and dispersal.

## Systematics and evolution

*C. intestinalis* is a member of the Tunicata (see Glossary). The Tunicata include the sessile ascidians, like *C. intestinalis*, as well as the free floating (pelagic) thaliaceans and larvaceans (see Glossary). Together with vertebrates and cephalochordates (see Glossary), tunicates make up the chordates. The defining feature of Chordata is the notochord, an anterior-to-posterior oriented rod of cells, which lies just below the spinal cord. In the first chordates, the notochord was likely to have conferred a biomechanical advantage to swimming larvae by providing them with stiffness and turgor, as it does for the *C. intestinalis* larva. Within the vertebrates, the notochord has an added role, acting as a signaling center for neighboring tissues of the developing embryo. Other morphological features uniting *C. intestinalis* (and other tunicates) to the chordates include a dorsal, hollow nervous system and a muscular post-anal tail (the anus of most ascidians, however, is re-positioned from the tail to the head in adult life).

Chordate phylogeny has been recently reordered (Delsuc et al., 2006; Singh et al., 2009), with tunicates and vertebrates now grouped as sister taxa within the chordates, where previously cephalochordates (e.g., amphioxus) and vertebrates were long thought to share a more-recent common ancestor (see for example, Schaeffer, 1987; Nielsen, 1995). This re-ordering has yielded new insights into the timing of the appearance of characters once believed to be unique to vertebrates. For example, neurogenic placodes (see Glossary), which in vertebrates give rise to the sensory structures of the head, including the olfactory and auditory systems, and neural crest cells (see Glossary), a key innovation in the elaboration of vertebrate cephalic structures (Gans and Northcutt, 1983), are both believed to have morphological antecedents in tunicates (Manni et al., 2001; Jeffery et al., 2004), indicating that these structures predate the divergence of tunicate and vertebrate lineages. Cephalochordates, by contrast, apparently lack neural crest-like cells or placode-like structures and may represent the basal chordate state, before these innovations occurred. Although some authors maintain that true placodes and neural crest remain vertebrate inventions (Schlosser et al., 2014), the newer phylogeny has highlighted the relevance of *C. intestinalis* in providing a window on the evolution of such traits.

Despite these conserved structures that unite the chordates, tunicates have several morphological features that diverge radically from their vertebrate cousins, including the adult body form, the absence of somites (see Glossary), hermaphroditic reproduction, and, curiously, the ability to synthesize cellulose (Kimura and Itoh, 1996; Matthysse et al., 2004; Nakashima et al., 2004). Cellulose production is thought to derive from the horizontal transfer of a prokaryotic cellulose synthase gene; the tunicate ancestor appears to have cobbled together the remainder of the cellulose biosynthetic pathway from its existing gene complement, as no other known plant or prokaryotic cellulose biosynthetic components have been identified in any tunicate. It has long been debated whether these divergent features were primitive (i.e., representative of the last common chordate ancestor) or derived (i.e., unique to tunicates). The finding that tunicates and vertebrates form a clade, with cephalochordates as the out-group, has shed new light on this issue. Cephalochordates lack the ability to make cellulose, have somites, and do not undergo a metamorphosis like most tunicates, arguing that many of the unique features of tunicates are derived (Swalla, 2006; Swalla and Smith, 2008). Likewise some tunicate genomic anomalies, such as degenerate Hox clusters (see Glossary), also appear to be derived (Ikuta et al., 2004).

## Habitat and life history

In the wild, *C. intestinalis* is a member of the marine biofouling (see Glossary) community, lining the hulls of boats and crowding the underwater surfaces of buoys and docks. It thrives even in the presence of the anthropogenic insults generated in a marina setting, such as effluent from boats, surface petroleum slicks and organic waste. *C. intestinalis* is cosmopolitan in distribution, found on both sides of the Atlantic and Pacific Oceans, and it has been reported in the oceans off Australia and South Africa (Therriault and Herborg, 2008b).

Two varieties of *C. intestinalis*, Type A and Type B, have been described. Type A is found on both sides of the Pacific, the Mediterranean and North Atlantic, and off the coasts of South Africa; Type B is found off the Atlantic coasts of Europe and North America (Caputi et al., 2007). The two types are known to overlap only in the English Channel and off the northern coast of France (Caputi et al., 2007; Nydam and Harrison, 2007; Sato et al., 2012). Reproductive barriers exist between the two, and attempts to rear fertile hybrids in the lab have been unsuccessful (Caputi et al., 2007); these facts alone suggest that A and B may, in fact, be properly viewed as two distinct species, not merely as sub-species. Nonetheless (and in apparent contradiction to laboratory observations), evidence of intermixing from the wild population within the shared range of A and B types shows that this reproductive isolation is not complete (Nydam and Harrison, 2011a). We therefore prefer to view A and B as sub-species even though they may be, at the same time, species in the making. This population provides a natural laboratory to investigate the mechanisms of speciation in action. For example, the proteins that mediate sperm-egg recognition have been reported to have evolved more rapidly than have other *C. intestinalis* proteins within the area of overlap, but still no faster than the rate at which these gamete-recognition proteins have changed in areas where only a single type is found (Nydam and Harrison, 2011b). The factors that have contributed to the divergence of these lineages bear greater examination, including differences in both life history (see Box 3) and genetics. Investigations into their genetic differences will likely be facilitated by the availability of separate assemblies for the genomes of Type A and Type B (Tassy et al., 2010). The existence of these subtypes also underscores the need to precisely determine the taxonomic status of any model system in order to clarify which features vary or are shared within and between species subtypes, particularly in the case of an organism like *C. intestinalis* where researchers rely almost entirely on wild-caught specimens.

10.7554/eLife.06024.005

Box 3. **Outstanding questions about the natural history of *C. intestinalis***

- What is the source of the high levels of genetic polymorphism found in *C. intestinalis?*

And how do polymorphisms vary between species subtypes or between more recently introduced populations compared to longer-established ones? Are high levels of polymorphism a more general character of tunicates or of broadcast spawners?

- What are the patterns of migration and invasion that have characterized *C. intestinalis*?

And what are the mechanisms of reproductive isolation between species subtypes (such as those reported between Types A and B)?

- What are the origins of cellulose synthesis, and how have tunicates integrated a functional cellulose synthase-encoding gene into their genome?

The production of cellulose is a defining feature of tunicates and may have influenced the group's life history as a sessile filter feeder: *C. intestinalis* cannot undergo metamorphosis if cellulose synthase function is blocked (Sasakura et al., 2005).

**DOI:** http://dx.doi.org/10.7554/eLife.06024.005

Although the place of origin of *C. intestinalis* is a matter of speculation (Therriault and Herborg, 2008a), they are thought to have extended their range through human activity, for instance, by travelling on the hulls of boats or by being carried in ballast water that is later discharged into the ocean. *C. intestinalis* was first reported in 1917 on the west coast of North America (Ritter and Forsyth, 1917). While its hardiness and adaptability to man-made environments makes *C. intestinalis* a nuisance—for example, accumulation on boat surfaces may add weight and drag to a vessel, and competition for space and food may hinder aquaculture (Ramsay et al., 2009)—these traits render these animals easily collectable for research purposes. As an immobile, indiscriminate filter feeder, *C. intestinalis* would appear to be an easy target for its predators which include sea stars, crabs, shrimp, nudibranchs and fish (Gullikesen and Skjaeveland, 1973; Carver et al., 2003; Dumont et al., 2009). However, its tough cellulose-containing tunic provides a level of protection and we speculate that the accumulation of toxins, in particular vanadium, a metal known to be found in high concentrations in ascidians (Michibata et al., 1991), could also serve as a deterrent.

The early development of *C. intestinalis*, from zygote to swimming larva, has been well described (Hotta et al., 2007). The embryo undergoes a regular pattern of development within a protective chorion, surrounded on the exterior by the finger-like gelatinous projections of the follicle cells. About 40 min after an egg is fertilized, an invariant series of cell divisions begins. At 112 cells (4.5 hr post fertilization), the embryo undergoes gastrulation (see Glossary). Neurulation soon follows (see Glossary), with the formation of the chordate-specific hollow, dorsal nervous system. About 18 hr after fertilization, a *C. intestinalis* larva hatches from its chorion and begins to search for a suitable spot to attach, and then undergo metamorphosis. Sensing gravity and light with two pigmented sensory organs found in its brain, the otolith and ocellus, respectively (see Figure 1A), the *C. intestinalis* larva generally swims up and away from light. As a result, larvae often settle on the undersides of docks and boats (Figure 1C), ropes and buoys—often conveniently within arms' reach of the surface. Similarly in the lab, we find that *C. intestinalis* swimming larvae readily attach to the surface of plastic petri dishes; hundreds can attach to a single large dish, which can then be transferred to the controlled conditions of a marine facility for rearing. The long-term culture of *C. intestinalis*, over multiple generations, is still best accomplished in a marine lab setting with access to unfiltered sea water, which provides these animals with their natural food source. However, *C. intestinalis* has also been reared with some success in facilities supplied with artificial sea water and re-hydrated food (Veeman et al., 2011).

*C. intestinalis* attach to a surface via the adhesive palps at their rostral tip (see Figure 1A), initiating a radical metamorphosis, the first event of which—the complete resorption of the tail—deprives the animal of it most recognizable chordate features. In the hours after settling, the animal develops a stalk, or stolon, at its attachment point, and its viscera are re-oriented within the body. Within a few days, a beating heart becomes visible. The mouth also opens, channeling water from the outside to the pharynx, and then through gill slits to right and left atrial chambers, and then back out into the surrounding sea water. Food is filtered from the incoming water, trapped on the pharyngeal mucus, a product of the endostyle (an organ thought to be homologous to the thyroid), and then directed to the esophagus and gut. Based on conserved features of vertebrates and cephalochordates, we can assume that in the ancestral chordate, the exit point of the gut, the anus, was at the ventral side of the tail. Because *C. intestinalis* reabsorbs its tail at metamorphosis, it must re-route this exit point. It does so through one of the atrial siphon cavities, usually the left. While anatomical and molecular evidence suggest that the atrial siphon primordia share a common origin with the primordia of the vertebrate ear (Manni et al., 2004; Mazet et al., 2005; Kourakis and Smith, 2007), the functional role of the atrial siphon in adult *C. intestinalis* is as a cloaca, a common exit point for waste, filtered sea water, sperm and eggs. The final topology of the adult animal is attained when, after a few weeks time, right and left atrial siphons fuse at the dorsal midline, yielding an animal with a single atrial siphon for outward-current flow, and a single, larger oral siphon, for inward-current flow (Figure 1B). After a few months time, the animals will be sexually mature and will live for up to 6 months (Satoh, 1994); in captivity, we find they live for as long as a year and a half.

## Reproduction and genetics

As an experimental organism, *C. intestinalis* has the advantage of being available and gravid year-round, although populations may wane during colder months (Berrill, 1937). At 3 months, individuals are typically 3–6 cm in length and are sexually mature. They are broadcast spawners, with several to hundreds of individuals simultaneously releasing eggs and sperm into the surrounding seawater, thereby increasing the chances of fertilization. Spawning occurs according to a light–dark cycle, cued by the arrival of daylight after several hours of darkness. We mimic this light–dark cycle in the lab for controlled spawning; *C. intestinalis* can be induced to spawn after less than 1 hr in darkness (Veeman et al., 2011).

*C. intestinalis*, like most other tunicates, are hermaphrodites, although recogniton molecules on the surface of the chorion limit the ability of animals to self-fertilize (Harada et al., 2008). While self-fertility is limited and not likely to play a large role in wild populations (Jiang and Smith, 2005), self-fertilization offers a convenient way for researchers to unmask recessive alleles responsible for developmentally noteworthy phenotypes. After eggs and sperm from a single individual are mixed, the resulting progeny can be screened during embryogenesis, larval life, or beyond, to identify morphological or behavioral variants (Sordino et al., 2008). In fact, wild *C. intestinalis* populations are a repository of genetic variability, so finding these mutants is not difficult. An analysis of *Ciona savignyi*, a closely related sister species to *C. intestinalis* that also has extensive polymorphism, has shown that its polymorphisms are not evenly distributed in the genome but are highest in intergenic regions and introns, as would be expected from purifying selection. It has been hypothesized that the high level of polymorphism is not due to an increased mutation rate, but rather to the large effective population size (Small et al., 2007).

The *C. intestinalis* genome has important differences compared to vertebrate genomes. In many cases, a developmental regulatory gene in *C. intestinalis* is represented by multiple orthologous genes from vertebrates. For example, the vertebrate genes *fgf8*, *fgf17*, and *fgf18* all share orthology—similarity by common descent—with the single *C. intestinalis* gene *fgf8/17/18*. During the vertebrate radiation, two rounds of whole-genome duplication likely occurred, producing four vertebrate genes for every one *C. intestinalis* gene, followed by subsequent gene loss and duplication events (Kasahara, 2007). This can simplify the study of gene interactions in *C. intestinalis* and facilitate experimental perturbations, such as gene knock-down. However, when the whole genome is considered, current best estimates show that *C. intestinalis* has ∼90% of the number of protein-coding genes as that found in the human genome (17,000 in *C. intestinalis* vs 19,000 in humans) (Satou et al., 2005; Tassy et al., 2010; Ezkurdia et al., 2014; Cunningham et al., 2015). The total genome size of *C. intestinalis*, however, is less than one twenty-fifth that of the human genome because of the reduction in non-coding gene sequence, such as introns, and because of the organization of about one fifth of its genes into operons (see Glossary; Satou et al., 2008). This small, compact genome offers various advantages to the researcher (Wang and Christiaen, 2012; Irvine, 2013).

## Conclusion

The tunicates—including *C. intestinalis*—are highly successful marine invertebrates. In most environments, *C. intestinalis* is considered to be invasive, and it can be so successful as to constitute a considerable fraction of the local biomass (Therriault and Herborg, 2008b; Ramsay et al., 2009). Many questions remain unanswered with regard to the sources and dates of its introduction, and regarding the relative contributions of human-mediated vs natural dispersion routes, and adaptations of various populations to local conditions (see also Box 3 for further outstanding questions). The widespread and easily collected populations of *C. intestinalis*, together with the genetic and genomic resources available for this organism, make this a ripe area for research.

The lifestyle adopted by the adult *C. intestinalis*, that of a sessile filter feeder, has served it well and has shaped its anatomy, physiology and embryology. Many of the features of *C. intestinalis* that at various times have been called primitive or basal, such as the simplicity of the embryo or the adult body form, are best seen as products of the animal's unique natural history. From a practical point of view, because of its inherently high levels of polymorphism and because of the many sites used to collect experimental animals worldwide, *C. intestinalis* researchers must remember that locally collected animals might be quite different to those used to generate the data found in *C. intestinalis* databases (see Box 2). With these unique features of *C. intestinalis* in mind, we can expect this relatively obscure marine model organism to contribute much to our understanding of basic chordate biology and to the history of the chordate clade.
